# Research progress on the mechanisms and intervention strategies of microbiota-gut-brain axis dysfunction under heat stress

**DOI:** 10.3389/fphys.2026.1897985

**Published:** 2026-07-31

**Authors:** Mo Niu, Fang Li, Zhiyi Hao, Jinying Dou, Zhongyan Xu, Keran Jia

**Affiliations:** Clinical Laboratory, Bethune International Peace Hospital, Shijiazhuang, Hebei, China

**Keywords:** heat stress, intestinal flora imbalance, microbiota-gut-brain axis, neuroinflammation, targeted therapy

## Abstract

Heat stress (HS), as an important environmental stressor in the context of global warming, profoundly affects body health by disrupting the Microbiota-Gut-Brain Axis (MGBA). This review provides a critical synthesis of the mechanisms underlying MGBA disruption under HS. HS initially induces gut microbiota imbalance, which subsequently disrupts the intestinal mechanical and immune barriers, thereby interfering with the bidirectional gut-brain signaling mediated by pathways such as the vagus nerve. Based on the above mechanisms, this review further explores MGBA-centered therapeutic strategies, including microbiota-oriented therapies such as probiotics, prebiotics, synbiotics, postbiotics, and fecal microbiota transplantation to reshape gut ecology, as well as the use of metabolites for targeted therapy. Finally, this article looks forward to future research directions in this field, emphasizing the exploration of molecular mechanisms and the search for key targets.

## 1 Introduction

Heat stress (HS) refers to the condition where the body’s heat load exceeds its physiological tolerance range without yet causing pathological damage. With the ongoing trend of global warming, the negative impacts of HS on human health and the livestock industry are becoming increasingly prominent (Hu et al., 2022). The gut microbiota plays an irreplaceable role in human health and is crucial for regulating normal gastrointestinal function and the pathological processes of diseases (Vicentini et al., 2021). Recognized as a key environmental hazard, HS can damage intestinal structure, disrupt microbial composition and diversity, and ultimately induce increased intestinal permeability and systemic inflammation (Jiang et al., 2021).

The microbiota is becoming increasingly important in human systemic diseases and brain health. Microbiota Gut Brain Axis (MGBA) as a new field is profoundly reconstructing our understanding of human physiological mechanisms (O’Riordan et al., 2025). MGBA is a bidirectional interactive network that integrates gut microbiota, gut environment, and central nervous system, regulating physiological functions and neurobehavior through neural, endocrine, and immune pathways (Cryan and Dinan, 2012). The gut microbiota is a complex collection of microorganisms that colonize the gastrointestinal tract, with bacteria as the dominant population, as well as symbiotic organisms such as fungi and viruses. The community structure and balance are dynamically regulated by multiple factors such as host genetics, environmental exposure, and diet. Intestinal bacteria break down food and convert it into nutrients for host utilization. The microbiota plays an important role in maintaining the physical and mental health of the host (Jeong et al., 2026). Traditional research has focused on the role of the epithelial barrier and innate immunity in maintaining intestinal homeostasis and resisting microbial invasion, but has long underestimated the contribution of the gut vascular barrier, despite the highly vascularized nature of the gut itself. Until recently, Gut-Vascular Barrier (GVB) has been established as a key secondary intestinal barrier structure (Mohamed Abdou et al., 2026). During this process, blood vessels serve as the core hub, coordinating the dynamic balance between the gut, peripheral tissues, and brain in terms of immune surveillance, substance exchange, and inflammatory response (Tran et al., 2026). The meningeal system consists of three consecutive membranes that tightly wrap around the surface of the brain and spinal cord within the skull and spinal canal. The dura mater, as a key interface layer, not only maintains a highly heterogeneous immune cell community under physiological homeostasis, but also possesses a special lymphatic drainage network (Marin-Rodero et al., 2025). Among them, immune cells include tissue resident macrophages, dendritic cells, and T lymphocytes involved in immune monitoring and neuroimmune communication (Fei et al., 2026). On one hand, the gut microbiota transmits signals to the central nervous system via neural, endocrine, and immune pathways; on the other hand, the central nervous system also regulates the gut microenvironment in return through neural control and immune responses, forming a complete neuro-endocrine-immune regulatory network (Karakan et al., 2021; Kesika et al., 2021). The vagus nerve, as a core component of the parasympathetic nervous system, plays an important regulatory role within the autonomic nervous system. The autonomic nervous system maintains physiological homeostasis by integrating mechanisms of endocrine regulation, motor coordination, and behavioral response. When the body encounters physiological or psychological stress, sympathetic excitation accompanied by parasympathetic inhibition occurs. This imbalance of neural tone can affect the function of the enteric nervous system through the neuro-gut axis. The enteric nervous system, by controlling intestinal smooth muscle contraction, regulating mucosal barrier permeability, and modulating the intensity of immune responses and levels of inflammation, ultimately achieves overall regulation of intestinal motility, permeability, and microbial community structure (Longo et al., 2023).

The MGBA plays a key role in the pathophysiological process of HS. This paper critically synthesizes existing evidence on the mechanisms of MGBA disruption under HS and evaluates MGBA-centered intervention strategies, highlighting knowledge gaps and future research priorities.

## 2 Mechanisms of MGBA disruption under HS

To cope with the negative impacts of environmental stress, various organisms have developed a set of rapid molecular response mechanisms to repair themselves and avoid persistent threats (Estruch, 2000). Positive and negative feedback mechanisms jointly maintain internal environmental homeostasis by regulating the interactions between microbial metabolic activities and host pathways (Lozupone et al., 2012).

### 2.1 Imbalance of gut microbiota and metabolic disorders

Studies have shown that after HS intervention, the α-diversity index in mice do not show significant changes, but there were significant differences in β-diversity, indicating substantial changes in the gut microbiota composition among different species (Wen et al., 2021; Lu et al., 2025). Moreover, similar phenomena have been observed in animal models of rabbit (Bai et al., 2021), chicken (Liu et al., 2022), and goat (Li et al., 2024) under HS conditions. Multiple studies have found that HS can cause changes in the gut microbiota structure of different animals (**Table 1**). These changes suggest that the effect of HS on the gut microbiota are species-specific.

**Table 1:** Effects of heat stress on gut microbiota composition in different animal species.

Animal	Microbiota changes (HS)(↑/↓ vs. control)
Mouse (Wen et al., 2021)	Lachnospiraceae bacterium 3-1 (↑); Candidatus Arthromitus sp. SFB-mouse-Japan and Lactobacillus murinus (↓)
Rabbit (Bai et al., 2021)	Firmicutes, Protobacteria, and Verrucomicrobiota (↑); Bacteriodota(↓)
Yellow-feathered broiler (Liu et al., 2022)	Ruminococcus, Bdellovibrio, and Serratia (↓)
Saanen goat (Li et al., 2024)	Prevotella and Bacteroidales (↑); Succinimonas and Ruminobacter (↓)
Grow-finishing pig (Xiong et al., 2020)	Proteobacteria (↑); Bacteroidetes (↓)
Dairy cow (Li et al., 2020)	Peptostreptococcaceae, Turicibacter, and Clostridium_sensu_stricto_1 (↑); Ruminococcaceae_UCG-005 and Rikenellaceae_RC9_gut_group and Bacteroides (↓)

Only major microbiota changes are listed. For specific abundance fold-changes and statistical information, please refer to the original literature. ↑: indicates significantly increased relative abundance; ↓: indicates significantly decreased relative abundance.

In addition to animal models, in recent years, Gould et al. found no differences in fecal microbiome composition, gastrointestinal barrier integrity, inflammation, or terminal temperature regulation indicators between 29 patients with exertional heat illness (EHI) and 29 patients without prior EHI history in their studies on the effects of HS on human gut microbiota, except for the Simpson index. However, it was found that the Firmicutes: Bacteroidota ratio decreased in individuals with heat intolerance (Gould et al., 2026). At present, the impact of HS on human gut microbiota may not be significant, and more evidence is needed to explore it.

Apart from changes at the taxonomic level, at the functional level, HS can alter the activity of metabolic pathways in the gut microbiota. Chen et al. (2018) found that under HS conditions, the expression of disease-related pathways, environmental adaptation pathways, and immune-related pathways in the gut microbiota of cows increased, while the expression of metabolic pathways decreased. Wen et al. (2021) found that in HS mice, pathways such as fatty acid biosynthesis, purine metabolism, and tyrosine metabolism were significantly enriched, among which the fatty acid biosynthesis pathway was significantly suppressed. Cui et al. (2019) Elevated levels of fluoride, lyxose 1, and D-galacturonic acid were found in HS pigs, all of which are associated with metabolites derived from the gut microbiota, most fatty acid metabolites increased, but arachidonic acid decreased; carbohydrate metabolites generally fluctuated. HS disrupts the gut microbiota, leading to metabolic reprogramming, causing bile acid imbalance, systemic changes in lipid and carbohydrate metabolism, and inflammatory reactions (Alkhoujah et al., 2026).

Although the studies summarized in **Table 1** consistently report HS-induced alterations in gut microbiota composition, cross-study comparisons remain challenging. First, HS protocols differ markedly in terms of temperature, duration, and humidity, making it unclear whether the reported microbial shifts reflect a universal response to heat load or protocol-specific artifacts. Second, methodological heterogeneity—including differences in sampling sites, DNA extraction kits, 16S rRNA variable regions, and bioinformatics pipelines—may contribute substantially to inter-study variability. Third, most studies report relative rather than absolute abundances, which can obscure true changes in microbial load. Importantly, the causal direction remains unresolved: does HS-induced gut dysbiosis drive MGBA dysfunction, or is dysbiosis a secondary epiphenomenon of altered host physiology? Gnotobiotic animal studies and fecal microbiota transplantation experiments that directly test whether heat-stress-associated microbiota can recapitulate MGBA phenotypes in unstressed recipients are urgently needed to establish causality.

### 2.2 Intestinal barrier damage

In addition to altering microbial composition, HS also directly damages the intestinal mechanical barrier, and these two effects may synergistically exacerbate each other. The intestinal mechanical barrier is jointly composed of the mucus layer and the tight junctions between mucosal epithelial cells (Ghulam Mohyuddin et al., 2022). The mucous layer of the intestine plays an important role in the intestinal barrier, and the goblet cells of the intestine secrete mucins composed of polysaccharides and proteins, forming a protective layer covering the intestinal epithelium. HS significantly downregulates the expression of mucin related genes, resulting in thinning or complete loss of the mucus layer (Fan et al., 2025). According to functional localization, mucins can be divided into transmembrane and secretory types: the former mediates intercellular adhesion processes, while the latter imparts key viscoelasticity to the mucus layer. In secretory mucin, gel forming mucin type 2 (MUC2) constitutes the main component of colonic mucus layer (de Ram et al., 2024). A study has found a decrease in the protein expression of MUC2 in the jejunum of HS yellow fatty broilers (Shao et al., 2026). Another study found that HS can reduce the expression of MUC2 gene in pig ileum (Oladele et al., 2021). However, under HS conditions, the detection of MUC-2 mucin related genes in cells showed that high temperatures under normal oxygen conditions can upregulate the transcription of MUC-2 genes, but this enhancing effect is weakened under hypoxia (Lian et al., 2021). Tissue hypoxia may be a key factor in HS inhibiting MUC2 expression *in vivo*. Tight junction, as a multi protein complex that regulates the cross epithelial transport of ions, small molecules, and water molecules, its core components include occludin, claudin, tricellulin, and junctional adhesion molecule (Ghulam Mohyuddin et al., 2022). Mechanistically, HS affects the expression of tight junction proteins by activating NF-κB-related signaling pathways, disrupting the integrity of the intestinal mechanical barrier (Zhang et al., 2024).

During HS, blood preferentially supplies tissues outside the gut such as muscle and brain, resulting in intestinal ischemia and hypoxia, and the permeability of intestinal barrier increases, prompting lipopolysaccharide (LPS) to enter the blood circulation. The leakage of LPS can stimulate the inflammatory response and release pro-inflammatory factors such as interleukin-6 (IL-6) (Vargas and Marino, 2016). LPS combined with myeloid differentiation factor 2 (MD2) and toll like receptor 4 (TLR4) to induce the dimerization of TLR4, then activate the transcription factor NF - κ B and initiate the cascade reaction of downstream pro-inflammatory cytokines (Roy et al., 2024). Intestinal hypoxia caused by HS disrupts the balance between reactive oxygen species (ROS) and the antioxidant defense system, thereby triggering epithelial tissue damage and inflammatory responses (Lambert et al., 2002). Under HS conditions, reduced blood supply and decreased food intake lead to oxidative stress and inflammatory responses, which further result in intestinal mucosal damage (Rostagno, 2020). In avian models, HS causes a significant decrease in villus height in the duodenum and ileum of broilers, while the number of goblet cells in the duodenum is significantly reduced (Wang et al., 2022).

### 2.3 Immune barrier disruption

The disruption of the mechanical barrier facilitates the translocation of microbial components, which in turn activates the intestinal mucosal immune system. The intestinal immune barrier is a key line of defense against intestinal pathogens, consisting of gut associated lymphoid tissue, various immune cells (such as T cells, B cells, dendritic cells, and macrophages), antimicrobial peptides released by Paneth cells, and immunoglobulin A synthesized by plasma cells (Damianos et al., 2025). Studies have shown that the number of Paneth cells in HS rats is significantly lower than that in the control group (Ducray et al., 2019). A study found that under HS conditions, the gene expression of Toll-like receptor 2(TLR2) and TLR4 in peripheral blood mononuclear cells of experimental animals was significantly upregulated, accompanied by enhanced inflammatory responses (Ju et al., 2014). In a study of broiler chickens, heat shock protein (HSP), as a stress-related pattern recognition receptor, can activate the TLR4 signaling pathway and induce the release of a large number of inflammatory factors through downstream MyD88 and TRIF pathways, leading to intestinal inflammation and tissue damage (Yang et al., 2024). HS exacerbates tissue inflammation and damage through the TLR4/NF-κB/NLRP3 signaling pathway (Dong et al., 2024). NLRP3 inflammasomes are activated in the brain to induce neuroinflammation (Xu et al., 2025). In addition, the JAK/STAT signaling pathway is also involved in the regulation of HS induced intestinal inflammation. Gene expression profiling analysis of the small intestine in HS rats showed significant changes in the expression of JAK-STAT pathway related genes, suggesting that this pathway plays an important regulatory role in heat stress-induced enteritis (Liu et al., 2014). HS leads to an imbalance in the inflammatory regulatory network through a dual regulatory mechanism. HS stimulation can trigger significant changes in the expression of inflammatory mediators. This is manifested as an upregulation trend of pro-inflammatory cytokines at the transcriptional and protein expression levels, and a significant inhibition of the expression level of anti-inflammatory cytokines. This stress state is accompanied by the activation of pro-inflammatory cytokine genes and the inhibition of anti-inflammatory cytokine genes (Wang et al., 2022). It also promotes the release of glutamate and upregulates the expression levels of metabotropic glutamate receptor 5 and excitatory amino acid transporter 3 in BV-2 microglia. Furthermore, HS stimulates the synthesis and secretion of pro-inflammatory cytokines, including interleukin-1β and interleukin-18 (Chen et al., 2024).

Inflammatory factors secreted by immune cells can migrate to the central nervous system by increasing the permeability of blood-brain barrier (BBB) (Huang et al., 2020). In addition, peripheral inflammatory factors can also transmit signals to the brain through the vagus nerve (Pavlov and Tracey, 2012). HS activates microglia and astrocytes in neuroinflammation (Huang et al., 2022a). Activation of glial cells can also induce the release of a large number of inflammatory factors. Finally, it causes neuroinflammation (Zhu et al., 2023).

### 2.4 HPA axis dysregulation

The hypothalamic-pituitary-adrenal (HPA) axis plays a crucial role in stress response (Dwyer et al., 2020). When HS destroys the oxidative balance of the body, excessive ROS can cross the BBB to activate the TRPV1 channel in the paraventricular nucleus of the hypothalamus, stimulate the secretion of corticotropin releasing hormone (CRH), trigger the HPA axis cascade reaction, and stimulate the synthesis and secretion of glucocorticoids (GCS). Finally, GCs is released into the systemic blood circulation (Perogamvros et al., 2012; Zhang et al., 2025). In addition, HS can also inhibit arginine synthesis to activate the HPA axis and reduce the levels of neurotransmitter signals such as γ - aminobutyric acid (GABA), brain derived neurotrophic factor (BDNF) (Fang et al., 2023).

It is worth noting that the interaction between the HPA axis and gut microbiota is not simply a one-way regulation, but exhibits a bidirectional feedback characteristic. On the one hand, the GCs released after HPA axis activation can directly regulate intestinal peristalsis, secretion, and visceral sensitivity, thereby changing the intestinal lumen environment, promoting the growth of some microbial communities and inhibiting others, leading to microbial dysbiosis (Yang and Hammamieh, 2025). Meanwhile, GCs can also downregulate the expression of tight junction proteins through glucocorticoid receptors (GR), impairing the integrity of the intestinal barrier (Zheng et al., 2026). On the other hand, gut microbiota can also reverse regulate HPA axis function. Aseptic animal experiments have shown that mice lacking gut microbiota have significantly increased GCs levels after stress, and the colonization of B. infantis can alleviate the HPA axis response in sterile mice. Colonizing enteropathogenic Escherichia coli will exacerbate the HPA axis response (Farzi et al., 2018). In addition, bacterial depletion can disrupt the circadian rhythms of the transcriptome and metabolome of the brain’s stress pathway, leading to abnormal GCs rhythms and excessive activation of the HPA axis during the sleep wake transition period (Tofani et al., 2025).

### 2.5 Summary

**Figure 1:**
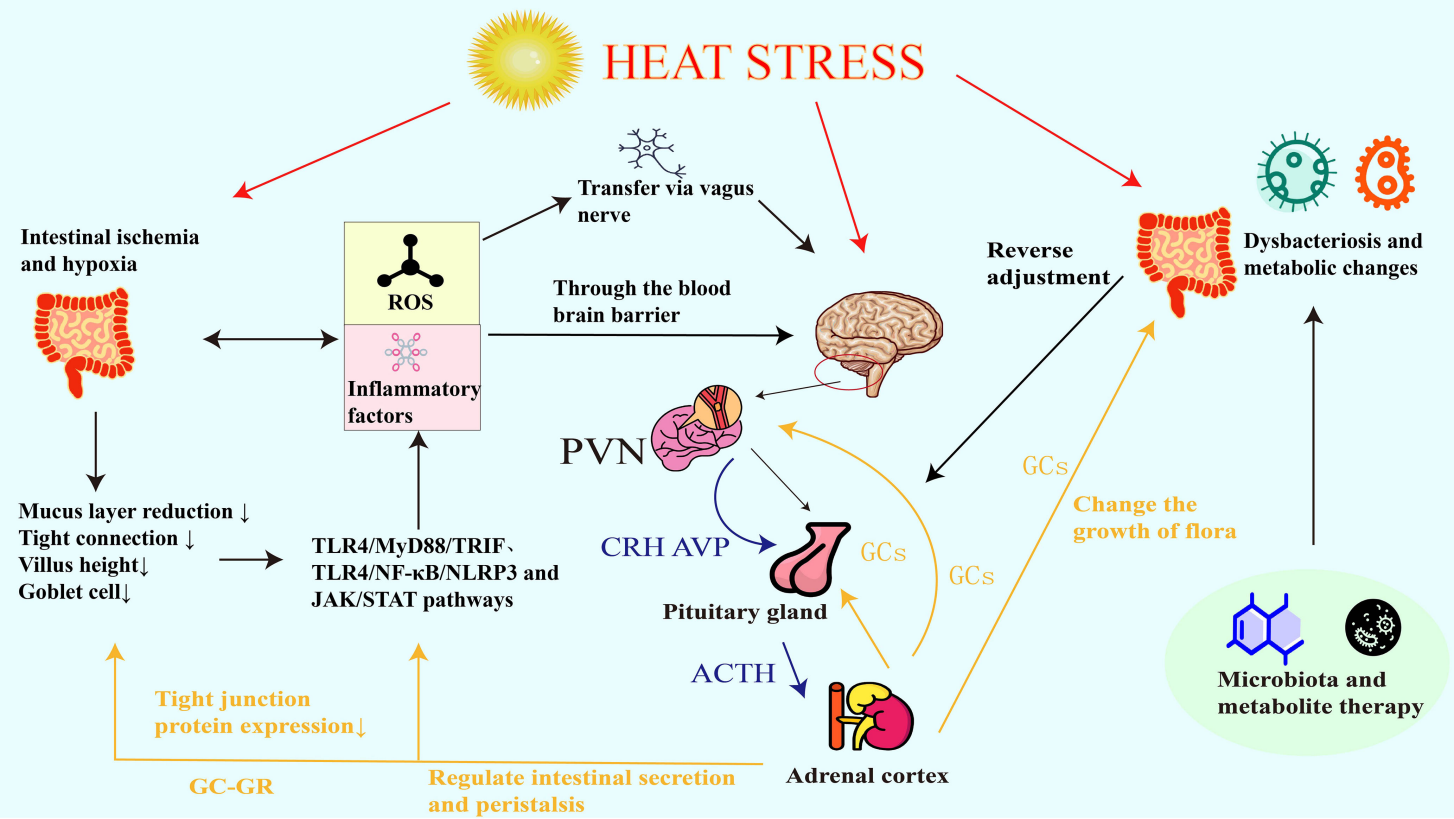
Pathophysiological cascade and feedback loops of MGBA dysfunction under heat stress. To facilitate understanding, the intestinal tract is divided into two parts, ROS and inflammatory factors can reach the brain through BBB and vagus nerve, but the effect of GCs on signaling pathway increases the release of inflammatory factors, ROS is unknown.

A critical limitation of the current literature is that the majority of evidence linking HS to neuroinflammation or behavioral changes via the MGBA remains correlational. For instance, while HS has been shown to activate microglia and increase hippocampal pro-inflammatory cytokines, whether these central effects are causally mediated by gut-derived signals—rather than by direct thermal effects on the brain or by systemic inflammatory mediators of non-gut origin—has yet to be experimentally dissected. Studies combining selective vagotomy, pharmacological blockade of specific microbial metabolite receptors, and gut-specific genetic manipulations under HS conditions would substantially strengthen causal inferences. **Figure 1** summarizes the disorder mechanism of HS on MGBA.

## 3 MGBA-targeted therapeutic strategies

### 3.1 Microbiota-oriented therapies

#### 3.1.1 Probiotics

HS often leads to a significant reduction in the abundance of beneficial bacterial genera such as Lactobacillus, making targeted supplementation of corresponding strains a reasonable intervention approach. Experimental verification has shown that compound probiotics containing Bacillus licheniformis, Bacillus subtilis, and Lactobacillus plantarum significantly enhance the growth performance of broiler chickens. By increasing the abundance of Lactobacillus and Bifidobacterium, these probiotics regulate intestinal microbiota, enhance the expression level of occludin to strengthen the barrier function, and effectively alleviate the adverse effects caused by HS (Song et al., 2014). Despite the potential demonstrated by probiotics in alleviating HS, their industrial application still faces challenges. The industrial development of probiotics encounters technical obstacles: metabolic activity attenuation and physiological function inactivation due to insufficient adaptability between production processes and strain characteristics; mechanical stress during processing, temperature control deviations, and fluctuations in cold chain storage and transportation conditions can all lead to the loss of strain viability (Mazziotta et al., 2023). Existing literature, including case reports, clinical trials, and experimental models, has elaborated on the potential theoretical risks of probiotics, including systemic infection, harmful metabolic activity, immune overactivation in susceptible populations, gene horizontal transfer, and gastrointestinal adverse reactions. However, expanded studies are needed to quantify the frequency and severity of these adverse events (Doron and Snydman, 2015).

#### 3.1.2 Prebiotics

Oligosaccharides such as fructooligosaccharides and galactooligosaccharides, which are difficult for the host to digest, activate downstream signaling pathways by binding to TLR4 receptors on the surface of epithelial cells and monocytes, thereby enhancing cytokine secretion levels. Their mechanism of action involves TLR4 ligand-mediated immune regulation, suggesting that these prebiotic components may affect host inflammatory responses by directly regulating innate immune receptors (Capitán-Cañadas et al., 2014; Ortega-González et al., 2014). Under periodic HS conditions, dietary supplementation with galactooligosaccharides significantly inhibited gene expression related to intestinal barrier function and inflammatory responses in the jejunum of broiler chickens, including heat shock protein genes, tight junction protein genes, inflammatory receptor genes, and cytokine genes. This alleviated the activation of stress signaling pathways in the small intestine caused by HS (Ringseis and Eder, 2022). In the intestinal environment, PH in particular plays a crucial role in determining the gut microbiota. Due to concerns about efficacy or safety, it is necessary to identify the stimulation of specific gut microbiota by prebiotics in order to develop prebiotics that promote human health (Chung et al., 2016).

#### 3.1.3 Synbiotics

The protective mechanism of synbiotics remains unclear, but its protective effect may be related to the combined use of probiotics and prebiotics (de Vrese and Schrezenmeir, 2008). Under HS conditions, supplementation with synbiotics alters the composition of the intestinal microbiota, with a significant reduction in coliform bacterial populations and coliform bacteria counts, and a significant increase in lactobacillus and bifidobacterium counts. In addition, synbiotic supplementation significantly increases the proportion of lymphocytes in broiler chickens and reduces the proportion of heterophils, resulting in a significantly lower heterophil lymphocyte ratio compared to the control group. The mechanism behind the decrease in the H/L ratio in this group may be related to the regulatory effect of plasma corticosterone concentration, indicating that the supplement may maintain homeostasis by regulating HPA axis activity (Mohammed et al., 2019). Due to the rich variety of probiotics and prebiotics, the vast number of possible combinations endows synbiotics with great potential in regulating the intestinal microbiota (Markowiak and Śliżewska, 2017).

#### 3.1.4 Postbiotics

Although probiotics are generally considered safe for healthy people, their safety is highly dependent on strain, dose, host and use environment (Churin et al., 2026). Consequently, there is a growing interest in emerging alternative preparations: postbiotics. Postbiotics refer to preparations made from non-living microorganisms and their components, which can confer health benefits to the host (Salminen et al., 2021). Supplementation of postbiotic preparations in broiler chickens under HS can effectively improve their pathological conditions. Supplementation with RI11 significantly alleviated growth inhibition caused by heat stress, while improving intestinal morphological structure and enhancing immune regulatory function. When using a compound postbiotic intervention protocol, the number of lactic acid bacteria significantly increased, while the proliferation of Enterobacteriaceae was inhibited, and intestinal villus development was significantly improved (Humam et al., 2019). Other studies have found that although postbiotics cannot alleviate the increase in inflammatory markers caused by HS, they can enhance energy utilization efficiency, water absorption rate, and intestinal permeability (Ríus et al., 2022).

Fecal microbiota transplantation (FMT): FMT is a method that achieves therapeutic purposes by transferring the intestinal microbial community from the feces of a healthy donor to the digestive tract of a patient. This technique is primarily used for the treatment of gastrointestinal diseases caused by pathogenic bacteria or opportunistic pathogens, by adjusting the structure of the patient’s intestinal flora to inhibit the abnormal proliferation of harmful microorganisms (Antushevich, 2020). Under HS conditions, FMT restructures the intestinal flora, increases the abundance of beneficial bacteria such as Citrobacter and Bifidobacterium, and reduces the abundance of harmful bacteria such as Clostridium difficile. FMT significantly restores the integrity of the colonic epithelium and promotes mucus secretion. At the same time, it downregulates the expression of key markers of the mitochondrial apoptosis pathway, such as Bax protein and Bak protein, inhibiting abnormal apoptosis of intestinal epithelial cells. Experimental data show that this therapy can effectively alleviate intestinal oxidative damage caused by HS (Liu et al., 2025). The application of gut microbiome research in clinical practice remains currently limited due to the complexity of the microbiota and its associated sequencing datasets, the difficulty in distinguishing correlation from causation, and the reliance on preclinical models with low human generalizability (Porcari et al., 2025). In order to compare the five microbiota oriented treatment strategies, their mechanisms, advantages, limitations and evidence levels are summarized in **Table 2**.

**Table 2:** Comparative summary of microbiota-targeted interventions against heat stress: mechanisms, advantages, limitations, and evidence levels.

Intervention strategy	Mechanisms	Advantages	Limitations	Evidence level
Probiotics	Direct supplement of active probiotics to maintain intestinal barrier	It has strong specificity and can be targeted for specific strains	The activity of the strain is easily affected by various factors; People with low immune level are vulnerable to infection (Mazziotta et al., 2023)	Animal experiments
Prebiotics	Direct regulation of immune response by binding to TLR4 receptors	Good stability, easy preservation, no risk of probiotic infection (Kuo, 2013)	Individual differences are large, and flora needs to be identified	Animal experiments
Synbiotics	The specific mechanism of the combination of probiotics and prebiotics is not clear	Probiotics and prebiotics synergism, the effect is better than a single intervention (Al-Habsi et al., 2024)	Various combinations, difficult to optimize	Animal experiments
Postbiotics	Direct supplement of inactivated bacterial components and/or metabolites	Good stability, convenient for storage and transportation (Żółkiewicz et al., 2020)	Difficult to standardize due to wide range of applications (Żółkiewicz et al., 2020)	Animal experiments
FMT	Transplant the whole intestinal microbial community of healthy donors to the digestive tract of patients, and comprehensively rebuild the microbial community ecology	Comprehensive effect, can restore the diversity and function of flora	The standardized processes of donor screening and bacterial solution preparation have not been unified; There are individual differences	Animal experiments

### 3.2 Metabolite-targeted therapy

#### 3.2.1 BHBA

BHBA, as an endogenous ketone body, can inhibit microglial pyroptosis and alleviate neuroinflammation by suppressing the STAT3-NLRP3-GSDMD signaling axis (Jiang et al., 2022). In the HS mouse model, BHBA intervention significantly reduces the density of Iba1-positive cells in the cerebral cortex, effectively inhibiting excessive activation of microglial cells and exerting a neuroprotective effect (Sui et al., 2024). Two other studies on mice also found similar findings: BHBA improved neuroinflammation in HS mice by inhibiting the activation of microglia and astrocytes, reducing synaptic associated proteins, and increasing dendritic spine density (Huang et al., 2022a, b). BHBA plays an important role in alleviating neuroinflammation in HS. However, an experiment based on pregnant cows found that calves born from high blood beta hydroxybutyrate cows had higher expression of stress genes, reduced brown adipose tissue mass, and decreased expression of thermogenic genes (Gai et al., 2025). Therefore, it is speculated that the high concentration of BHBA in the parent may affect the offspring’s ability to withstand high temperatures. This suggests that if BHBA is used as a preventive measure against heat stress, more evidence is needed to explore the safety of dosage.

#### 3.2.2 GABA

GABA is produced from L-glutamic acid catalyzed by glutamate decarboxylase (GAD) (Cui et al., 2020). Supplementation of GABA in HS cows can effectively enhance their immune response function and antioxidant defense mechanism (Cheng et al., 2016). Other studies have shown that by supplementing cows with GABA, rectal temperature can be significantly reduced, dry matter intake and milk yield can be increased, and milk protein and lactose concentrations can be enhanced, thereby effectively alleviating the effect of HS (Cheng et al., 2014).

#### 3.2.3 DMG

DMG effectively improves the bioavailability of key nutrients in feed by optimizing the intestinal emulsification environment (Chalvatzi et al., 2020). DMG intervention significantly increases the abundance of short-chain fatty acid-producing bacteria in the intestine, restores tryptophan metabolic balance, promotes the secretion of anti-inflammatory factor IL-10, and simultaneously inhibits the production of pro-inflammatory factors IL-6 and IL-8, thereby reducing intestinal inflammatory response by suppressing the activity of the NF-κB signaling pathway. Additionally, DMG repairs HS-induced intestinal mucosal mechanical barrier damage by upregulating the expression of tight junction proteins Claudin-1 and Occludin (Wang et al., 2022).

As a signaling molecule, bile acids (BA) are synthesized in the liver using cholesterol as a raw material. Their metabolic transformation is regulated by the gut microbiota, and changes in bile acids in turn shape the composition of the microbiota (He et al., 2026). Farnesoid X receptor (FXR) and Takeda G-protein coupled receptor 5 (TGR5) play important roles as BA receptors in maintaining intestinal substance metabolism and movement (Wang et al., 2025). It was found that BA supplementation could improve the average daily gain, feed conversion rate and antioxidant capacity of HS broilers (Yin et al., 2021). Many studies have found that BA supplementation plays a positive role in regulating the immune function of broilers (Bansal et al., 2020; Hu et al., 2024; Wang et al., 2025). These findings indicate that BA as a nutritional strategy has certain potential in alleviating heat stress. Tryptophan (TRP) metabolism is related to the gut brain axis, which mainly includes three pathways, namely kynurenine pathway (KP), 5-hydroxytryptamine (5-HT) pathway and indole metabolism pathway (O’Mahony et al., 2015; Yan et al., 2024). Indole is a key intercellular and bacterial signal molecule. The representative substance indole-3-acetic acid (IAA) can maintain the integrity of intestinal epithelium, strengthen the barrier function and play an anti-inflammatory role by activating the aromatic hydrocarbon receptor (AHR). Its beneficial effects also extend to the central nervous system and metabolic regulation. The regulation of IAA on intestinal homeostasis, barrier integrity, immune response and lipid metabolism involves AHR dependent and AHR independent signaling pathways, which makes it a potential candidate for intervention in gastrointestinal and nervous system diseases (Roager and Licht, 2018; Shaheen et al., 2025). It was found in melatonin treated goats that melatonin alleviated heat stress by maintaining mitochondrial integrity and redox homeostasis (Yang et al., 2025). 5-hydroxytryptamine (5-HT), also known as serotonin, is not only a monoamine neurotransmitter in the central nervous system, but also a key gastrointestinal signaling molecule, which is responsible for transmitting signals from the gut to internal and external neurons (Mawe and Hoffman, 2013). Many experiments have found that probiotics regulate Trp metabolism by increasing the abundance of beneficial bacteria in the intestine, regulating neurotransmitters and increasing the level of 5-HT, thus affecting the central nervous system and immune system (Cheng et al., 2026). In addition, the study found that TRP supplementation could effectively prevent HS induced oxidative damage and mitochondrial dysfunction by regulating antioxidant status and up regulating the expression of genes related to mitochondrial function in broilers (Ouyang et al., 2022).

### 3.3 Potential therapeutic strategies

Currently, there is no research indicating that exogenous short-chain fatty acids (SCFAs) have a protective effect on HS-induced MGBA dysfunction. However, given their known biological activities, this section will discuss their theoretical potential. SCFAs are key bioactive molecules produced by the metabolism of gut microbiota, capable of penetrating the BBB and interacting with neuroimmune cells. SCFAs can inhibit the release of pro-inflammatory factors by regulating THP-1-induced microglia (Wenzel et al., 2020). Not only can SCFAs cross the BBB, but they also play a crucial role in maintaining the integrity of its barrier function (Silva et al., 2020). Studies have found that butyrate treatment can significantly reduce BBB permeability and enhance occludin protein expression in the frontal cortex and hippocampus (Braniste et al., 2014). However, before becoming a therapeutic measure, SCFAs still faces many challenges: rapid intestinal absorption, difficult dose optimization, limited targeted delivery and significant individual differences. For example, butyrate is metabolized rapidly in the intestines and has a bad smell, which leads to low oral absorption (Cao et al., 2024). It is rapidly absorbed as an energy substance in the proximal intestine, making it unable to reach the distal colon (Sakata, 2019). The results showed that the same SCFAs had different effects at different doses, and different SCFA had different effects at the same dose (Alzubi et al., 2025). Therefore, the dose optimization of SCFAs has become a key issue. In addition, different routes of Administration (oral, intravenous, rectal, etc.) will affect the pathway and mechanism of action of SCFAs into the body, and different species have different reactions to SCFA (Kong et al., 2021). Individual differences also limit the development of SCFAs as a therapeutic measure. Therefore, SCFAs is currently still a theoretical strategy rather than an evidence-based strategy.

## 4 Discussion

This review is a narrative synthesis rather than a systematic review; literature selection was purposive rather than exhaustive, and formal quality assessment of individual studies was not conducted. Therefore, the conclusions drawn reflect the authors’ interpretation of the available evidence and should be corroborated by future systematic and quantitative meta-analyses.

HS can directly affect the abundance of microbial communities, inhibit fatty acid synthesis, reduce arachidonic acid, cause fluctuations in carbohydrate metabolism, lead to bile acid imbalance and metabolic reprogramming; Meanwhile, blood redistribution leads to intestinal ischemia and hypoxia, not only directly downregulating the expression of MUC2 mucin, but also disrupting tight junction proteins by activating the NF - κ B pathway, increasing the permeability of the intestinal barrier. After LPS leakage, it binds to MD2 and TLR4, activates NF - κ B, and releases pro-inflammatory factors such as IL-6. HS can induce the release of inflammatory factors and inhibit anti-inflammatory factors through the TLR4/MyD88/trif, TLR4/NF-κB/NLRP3, and JAK/STAT pathways, leading to intestinal inflammation. Peripheral inflammatory factors induce neuroinflammation by increasing the permeability of the BBB and vagus nerve inputs, activating microglia and astrocytes, promoting glutamate release, upregulating metabolic glutamate receptor 5 and excitatory amino acid transporter 3, and synthesizing interleukin-1 β and interleukin-18 to reach the central nervous system. At the neuroendocrine level, HS induced oxidative imbalance leads to excessive ROS crossing the BBB, activating the TRPV1 channel in the paraventricular nucleus of the hypothalamus, stimulating CRH secretion and triggering the HPA axis cascade reaction, promoting the massive release of GC, inhibiting arginine synthesis, weakening BHBAd and BNDF signaling, and further activating the HPA axis; The released GCS further regulates intestinal peristalsis, secretion, and visceral sensitivity, downregulating tight junction proteins through glucocorticoid receptors, exacerbating microbiota imbalance and barrier damage, thereby forming a cycle of microbiota disorder, barrier disruption, immune inflammation, neuroinflammation, and HPA axis hyperactivity. The whole process is not a single line advancement, but multiple lines running in parallel and influencing each other.

Despite the promising research findings, it is important to acknowledge several limitations and controversies. Firstly, most studies are based on animal models under acute heat exposure conditions, which may not fully reflect the situation of chronic, low-level heat exposure in humans. Secondly, the translational potential of microbial-targeted therapy is hindered by individual baseline gut microbiota variability and the lack of standardized protocols. Thirdly, as most evidence is correlational, the causal relationship between specific microbial groups and neurobehavioral outcomes under heat exposure remains to be established. Addressing these gaps is crucial for clinical application.

Future research should shift from describing phenomena to dissecting molecular mechanisms, exploring the connections between specific microbial metabolites (such as short-chain fatty acids and tryptophan metabolites) and the regulation of the HPA axis as well as the activation of microglia. It is crucial to identify key regulatory targets, such as the STAT3-NLRP3 axis and the NF-κB pathway, for drug development. Additionally, early biomarkers should be explored to serve as early warning indicators for HS-induced MGBA dysfunction.
